# Assessing ChatGPT’s Role in Sarcopenia and Nutrition: Insights from a Descriptive Study on AI-Driven Solutions

**DOI:** 10.3390/jcm14051747

**Published:** 2025-03-05

**Authors:** Özlem Karataş, Seden Demirci, Kaan Pota, Serpil Tuna

**Affiliations:** 1 Department of Physical Medicine and Rehabilitation, Akdeniz University, Antalya 07070, Turkey; 2 Department of Neurology, Akdeniz University, Antalya 07070, Turkey; sdndemirci@yahoo.com; 3 Department of Orthopaedics and Traumatology, Akdeniz University, Antalya 07070, Turkey

**Keywords:** sarcopenia, ChatGPT, nutrition, artificial intelligence, large language models, patient education

## Abstract

**Background:** Sarcopenia, an age-related decline in muscle mass and function, poses significant health risks. While AI tools like ChatGPT-4 (ChatGPT-4o) are increasingly used in healthcare, their accuracy in addressing sarcopenia remains unclear. **Methods:** ChatGPT-4’s responses to 20 frequently asked sarcopenia-related questions were evaluated by 34 experts using a four-criterion scale (relevance, accuracy, clarity, Ccmpleteness). Responses were rated from 1 (low) to 5 (high), and interrater reliability was assessed via intraclass correlation coefficient (ICC). **Results:** ChatGPT-4 received consistently high median scores (5.0), with ≥90% of evaluators rating responses ≥4. Relevance had the highest mean score (4.7 ± 0.5), followed by accuracy (4.6 ± 0.6), clarity (4.6 ± 0.6), and completeness (4.6 ± 0.7). ICC analysis showed poor agreement (0.416), with Completeness displaying moderate agreement (0.569). **Conclusions:** ChatGPT-4 provides highly relevant and structured responses but with variability in accuracy and clarity. While it shows potential for patient education, expert oversight remains essential to ensure clinical validity. Future studies should explore patient-specific data integration and AI comparisons to refine its role in sarcopenia management.

## 1. Background

Sarcopenia is an important syndrome frequently observed in the elderly population, characterized by a decrease in skeletal muscle mass and physical function associated with aging, and it has multidimensional effects [1]. This condition leads to serious outcomes such as physical disability, loss of independence, reduced quality of life, and an increased risk of mortality, thus imposing a significant burden on both individuals and healthcare systems [2,3]. In the development of sarcopenia, in addition to biological processes such as age-related inflammation, oxidative stress, and endocrine changes, inadequate protein and energy intake, micronutrient deficiencies, and poor dietary quality are also among the fundamental risk factors [3,4]. Therefore, a comprehensive understanding of the underlying mechanisms of sarcopenia is critical for devising both preventive and therapeutic strategies.

Rapid advancements in digital technologies have steadily increased the use of innovative applications such as artificial intelligence (AI) and large language models (LLMs) in healthcare services [5,6]. Thanks to text processing, deep learning algorithms, and natural language processing techniques, LLMs like ChatGPT can offer fast, accessible, and personalized solutions in patient education, clinical decision support, research, and information access [7]. The number of studies investigating the potential use of these models in medical education and patient counseling is growing daily. For instance, Miner et al. (2016) demonstrated that a smartphone-based conversational agent could provide coherent responses to health-related queries, and Laranjo et al. (2018) reported on the effectiveness of conversational agents in enhancing patient engagement in healthcare, including aspects of nutritional counseling [8,9]. The number of studies investigating the potential use of these models in medical education and patient counseling is growing daily. However, these models are also noted to have limitations, such as not fully comprehending complex medical conditions, harboring certain biases, and potentially diverting individuals from necessary in-person medical consultations [7,10]. Although the number of studies on the potential use of these models is increasing daily, especially in medical education and patient counseling processes, they have not been sufficiently examined for their accuracy with respect to multidimensional conditions that are critically important for older adults, such as sarcopenia. This research gap underscores the need to evaluate the reliability of the information these models provide on sarcopenia and nutrition, as well as to investigate how this information could contribute to patients’ understanding and management of sarcopenia, a common condition, and related nutritional concerns.

This study aims to evaluate ChatGPT-like LLMs’ responses regarding sarcopenia and nutrition in terms of scientific accuracy, comprehensibility, applicability, and readability, in light of the clinical guidelines. It is believed that the findings will shed light on the more effective and evidence-based use of AI-based tools in clinical practice and patient education. Additionally, these findings may help clinicians determine the appropriate role of AI-based tools in patient education and decision-making, facilitating better access to reliable health information for older adults at risk of sarcopenia.

## 2. Methods

**Figure 1:**
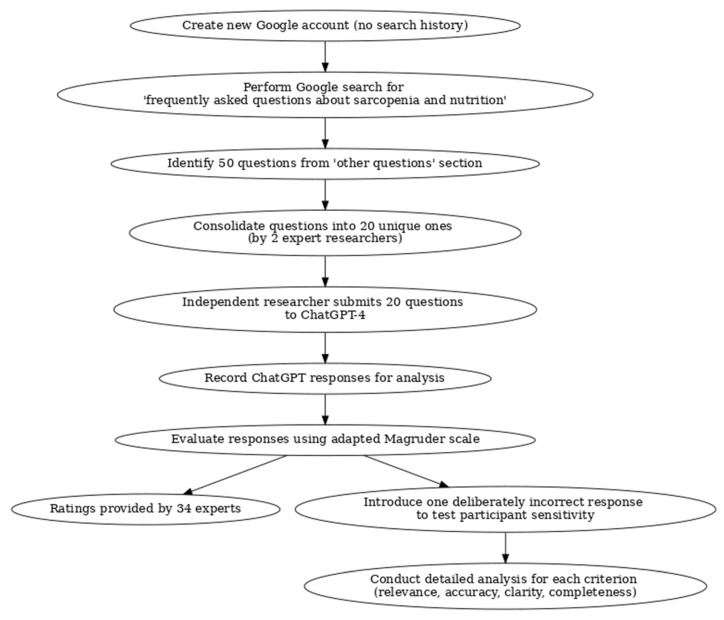
Flowchart illustrating the study design and methodology, including question identification, response evaluation, and analysis.

This study aimed to evaluate ChatGPT-4’s (ChatGPT-4o, OpenAI, San Francisco, CA, USA) responses concerning sarcopenia and nutrition. Questions were gathered from two primary sources: those frequently asked by patients in our clinic and those obtained through a Google search. Google is the most widely used search engine by the general public for health-related queries; therefore, the questions obtained via Google search reflect real-world inquiries. Additionally, by using a newly created Google account with no search history, the influence of personalized results has been minimized, making the questions more representative for general patient education purposes. As part of the study, a new Google account with no search history was created, and a search was performed using the keywords “frequently asked questions about sarcopenia and nutrition”. From the “other questions” section in the Google search results, a total of 50 questions were identified. These questions were reviewed by two researchers experienced in the field of sarcopenia and nutrition, and similar or repeated statements were consolidated into 20 unique questions. (The detailed steps of the study design are summarized in Figure 1). An independent researcher, who did not participate in the question development process, was assigned the task of submitting these questions to the ChatGPT platform. This method was chosen to ensure impartiality during the evaluation process. The questions were asked on ChatGPT-4 in November 2024 at different time intervals to minimize sequential response bias. ChatGPT’s responses were recorded and prepared for analysis. Since this study did not involve human participants and was based solely on the analysis of an online tool, no ethical committee approval was required.

The obtained responses were evaluated using a scale adapted from the method by Magruder et al. [9]. Of the six original criteria in the scale, four were included in the study: relevance, accuracy, clarity, and completeness. Relevance assessed whether the response was directly related to the question, accuracy examined the alignment of the response with scientific information, clarity judged whether the response was clear and understandable, and completeness evaluated whether the response contained all necessary information. In this study, the “evidence-based content” criterion was excluded because ChatGPT does not provide explicit references for each response. Moreover, prior research has demonstrated that AI models, including ChatGPT, may generate hallucinated references—fabricated citations that do not correspond to real scientific sources. This phenomenon, commonly reported in large language models, poses a challenge for evidence verification and reliability in medical applications. This introduces significant verification challenges, potentially compromising the reliability of an evidence-based evaluation. Therefore, we adapted the evaluation framework by excluding this criterion while preserving the core structure of Magruder et al.’s approach to ensure that the assessment remained rigorous and aligned with the specific objectives of our study [11].

The answers were rated using a scoring system ranging from 1 (low) to 5 (high) by 34 experts who had at least five years of experience in the field of sarcopenia and nutrition. During the evaluation process, the experts assessed the answers based on their clinical expertise and the guidelines provided by the European Working Group on Sarcopenia in Older People (EWGSOP) and the American Society for Nutrition (ASN). These guidelines served as reference standards to ensure that the assessment aligned with established medical knowledge [2,12]. A detailed analysis was conducted for each criterion to measure the appropriateness and quality of the responses.

## 3. Statistical Analysis

The data were analyzed using IBM SPSS version 23.0 (SPSS Inc., Chicago, IL, USA). Descriptive statistics were presented as counts, percentages, mean ± standard deviation, and median (minimum; maximum). After 34 evaluators scored the answers to 20 questions based on four criteria, the average score for each question was calculated using the scores provided by all evaluators. This process was repeated separately for each criterion: relevance, accuracy, clarity, and completeness. As a result, a single evaluation score was obtained for each question under each criterion. To compare the scores across all criteria for all questions, the Wilcoxon signed rank test was used to determine if there were significant differences between the scores. Interrater reliability (IRR) was assessed using the intraclass correlation coefficient (ICC) to evaluate agreement (two-way mixed effects model, absolute agreement, multiple raters). ICC values less than 0.50 indicate poor agreement, values between 0.50 and 0.75 indicate moderate agreement, values between 0.75 and 0.90 suggest good agreement, and values greater than 0.90 indicate excellent agreement [13]. A *p*-value of <0.05 was set as the level of statistical significance for this study.

## 4. Results

**Figure 2:**
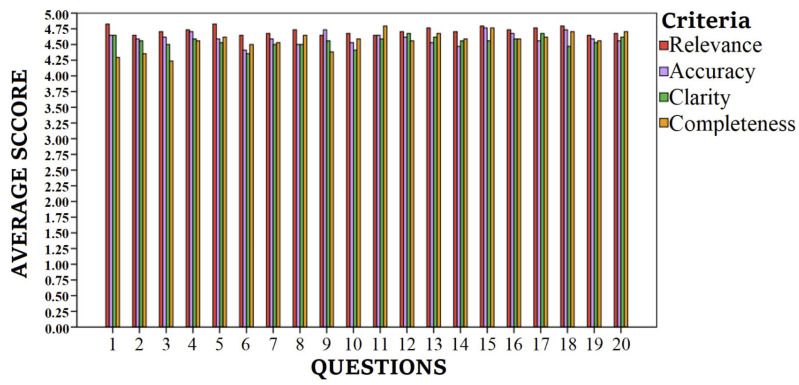
The average question responses (Q1–Q20) for four criteria.

Table 1 lists the 20 most frequently asked questions about sarcopenia and nutrition, evaluated by 34 respondents based on four criteria: relevance, accuracy, clarity, and completeness. Each question received an average score, a median score (ranging from 2.0 to 5.0), and the percentage of evaluators rating the questions highly (≥4 across all criteria). All questions received high median scores (5.0), and a high percentage of evaluators rated them positively (≥90% for almost all questions). Figure 2 illustrates the average scores of the 20 questions (Q1–Q20) across four criteria: relevance, accuracy, clarity, and completeness. The scores demonstrate consistent high ratings across all questions, with minimal variation between criteria. Relevance consistently received the highest scores, followed by accuracy, completeness, and clarity.

The highest mean scores were observed in relevance (4.7 ± 0.5), followed by accuracy (4.6 ± 0.6), clarity (4.6 ± 0.6), and completeness (4.6 ± 0.7). Significant differences were found between relevance vs. accuracy and relevance vs. clarity (both *p* < 0.001) and between relevance vs. completeness median scores (*p* = 0.001). No significant differences were observed between accuracy vs. clarity (*p* = 0.054), accuracy vs. completeness (*p* = 0.642), and clarity vs. completeness median scores (*p* = 0.586) (Table 2). When assessing IRR, only the completeness dimension demonstrated moderate agreement (ICC = 0.569; *p* < 0.001). Relevance (ICC = −0.104; *p* = 0.684), accuracy (ICC = 0.127; *p* = 0.208), and clarity (ICC = 0.022; *p* = 0.417) all showed poor agreement (ICC < 0.5), indicating poor reliability. Similarly, the overall agreement among evaluators was classified as poor (ICC = 0.416) (Table 3).

## 5. Discussion

In this study, we aimed to evaluate the quality of ChatGPT responses to common questions related to sarcopenia and nutrition among individuals in the general population and to examine whether these responses possess an acceptable level of adequacy. To achieve this goal, we utilized expert opinions and conducted a survey based on four predefined criteria: relevance, accuracy, completeness, and clarity. Overall, it was observed that the responses generated by ChatGPT received relatively high scores across all criteria, with all questions averaging ≥4 points. The majority of the questions’ responses (17 out of 20) received a rate of respondents rating ≥4 for all criteria consistently above 90%, reflecting high agreement on their quality. However, the following three questions had a slightly lower rating, with 90% of respondents scoring them ≥4: “How much daily protein do I need, and from which foods should I obtain these proteins?”, “Are protein powders or amino acid supplements recommended for sarcopenia? When and how should I use them?”, and “Does limiting carbohydrate intake increase the risk of sarcopenia, or is it more important to focus on protein?” The mean and median values in Table 2 are quite high and close to each other. The standard deviations are also very small, indicating that evaluators generally provided similar scores. This situation suggests that the scores are concentrated within a narrow range, and due to the low variance, the ICC value has also decreased. Additionally, the inability of the evaluation criteria to create a wider distribution may have also contributed to the low ICC value. Low ICC does not always indicate inconsistency among evaluators. Evaluators may be highly consistent, but if they assign scores within a narrow range, the ICC value can still be low [13,14].

It was observed that ChatGPT’s responses to general qualitative questions demonstrated higher overall performance compared to its responses to follow-up or symptom-focused questions. These findings revealed that among the four criteria, the highest average score was obtained in relevance, followed by accuracy, completeness, and clarity (Table 2). Statistical comparisons indicated significant differences between relevance and the other dimensions (*p* < 0.05). This suggests that ChatGPT generally remains on topic and provides contextually appropriate information that directly addresses user inquiries. Although accuracy scores were also high, they were statistically lower than relevance; this indicates that while ChatGPT’s responses are pertinent, there is some variability concerning scientific accuracy and compliance with current medical guidelines. However, no significant differences were found among accuracy, completeness, and clarity (*p* > 0.05), indicating generally consistent yet somewhat variable performance across these three categories.

The potential applications of AI-supported systems, particularly natural language processing models like ChatGPT, in the healthcare sector have been intensely debated in recent years. Thanks to their ability to rapidly access large datasets, analyze this data, and provide understandable and consistent responses, such models are considered to potentially play significant roles in medical education, clinical research, and patient care [15]. As medical knowledge rapidly expands and clinical decision-making processes become increasingly complex, having a reliable and easily accessible source of information can offer significant advantages to both healthcare professionals and patients.

The results of this study are partially consistent with similar studies in the literature. In a previous study focusing on general nutrition topics [16], it was reported that ChatGPT was more successful in most questions compared to responses from dietitians. However, in our study, a specific clinical context such as sarcopenia was considered, and more complex questions were examined. The ability of ChatGPT to provide recommendations tailored to specific patient groups was examined within the context of sarcopenia in our study. Although previous studies have demonstrated ChatGPT’s potential in generating meal plans for chronic diseases such as type 2 diabetes mellitus, our study highlights its limitations in adapting recommendations to sarcopenia-specific needs. Unlike conditions where general dietary guidelines are well-established, sarcopenia management requires tailored protein intake, resistance training considerations, and micronutrient optimization, factors that ChatGPT does not consistently address [17].

One possible reason for the variability in ChatGPT’s patient-specific recommendations is its inability to incorporate individual clinical history, biomarkers, and functional assessments. Unlike human experts who adjust dietary guidance based on comorbidities and lifestyle factors, ChatGPT relies on generalized text-based knowledge, which may not fully account for the nuances of sarcopenia management.

A recent study examined ChatGPT’s ability to create personalized diet plans for hypothetical individuals with chronic diseases such as obesity, cardiovascular disease, and type 2 diabetes mellitus. The research evaluated aspects of the meal plans, including energy intake, accuracy of nutrient content, and variety. The results revealed that the plans generated by ChatGPT were generally diverse; however, they could exhibit deviations from target energy requirements of up to 20% [18]. Nevertheless, it was observed that these deviations significantly decreased when the target energy requirements were clearly specified. For example, for ChatGPT version 3.5, deviations decreased from 19.6% to 17.3%, while for version 4.0, this rate reduced from 27.7% to 3.4% [19].

These findings indicate that detailed information provided by the user plays a critical role in enabling ChatGPT to create more precise and purpose-driven plans. Strong scores in relevance and medium to high scores in accuracy, clarity, and completeness suggest that ChatGPT can serve as a complementary tool for patient education. For instance, healthcare professionals can use ChatGPT to generate preliminary informational materials on sarcopenia and nutrition, which they can then review and tailor to individual needs. This approach can optimize the time spent on patient counseling and allow clinicians to address more complex or nuanced topics. However, it is crucial to also consider the clinical and ethical risks associated with AI-supported recommendations. For instance, ChatGPT’s potential to generate inaccurate, incomplete, or misleading information owing to issues such as hallucinations can pose significant risks to patient safety. Therefore, ChatGPT should be used as a complementary tool rather than a replacement for clinical expertise, and all AI-generated content must be subject to expert validation.

With the impact of the digital age, the rate of data production is continuously increasing worldwide. Medical data is also affected by this rapid growth, presenting both opportunities and challenges for healthcare professionals and researchers. Effective processing and interpretation of this data have become possible with the assistance of artificial intelligence technologies [20]. ChatGPT, developed by OpenAI, stands out as a tool that can guide not only general information seekers but also individuals seeking medical information [21,22]. AI-supported tools are increasingly being used in patient monitoring, diagnostic processes, and the creation of personalized treatment plans. However, the rapid increase in data volume presents not only opportunities but also challenges [23]. While healthcare professionals find it difficult to make sense of such large amounts of information, AI technologies have the potential to filter and process this information, making it more accessible. Although AI-supported systems are powerful tools for data processing and analysis, the strength of human–machine interaction plays a key role in the success of these systems. Models like ChatGPT, with their user-friendly interfaces, can help not only healthcare professionals but also patients understand complex information.

Despite the many advantages provided by ChatGPT, this technology still has significant limitations. ChatGPT operates based on a predetermined training dataset, lacks individual context or emotional insight, processes information without independently verifying the accuracy of its sources, and lacks direct control mechanisms. Studies have shown that evidence regarding the accuracy and reliability of ChatGPT in different clinical fields is limited [24]. Furthermore, a large portion of these studies have focused on ChatGPT’s general medical potential, requiring more detailed examinations for specific medical areas. Additionally, ChatGPT’s responses may be influenced by dietary guidelines and research predominantly published in English-language sources, which are often based on nutritional standards from Europe and North America. This may result in limited representation of region-specific dietary patterns, such as traditional Asian, Mediterranean, or plant-based diets more commonly adopted in certain cultures. These ethical concerns ranging from data bias and accountability to transparency and patient privacy further underscore the necessity for rigorous expert oversight when utilizing AI-generated recommendations. This limitation highlights the importance of expert validation when using AI-generated responses for patient education [25]. Moreover, AI-generated responses are prone to hallucinations producing non-existent or misleading recommendations, particularly in medical contexts. Unlike human experts, ChatGPT lacks real-time access to updated scientific literature, meaning that its responses may not align with the latest clinical guidelines. Therefore, future AI models should incorporate mechanisms to minimize misinformation and improve contextual adaptation to diverse populations and dietary habits [26,27]. In the fields of sarcopenia and nutrition, research on the potential of AI-supported tools to provide accurate and meaningful information is limited [26,28]. As access to online information becomes widespread, evaluating the reliability of this information and directing individuals correctly becomes increasingly critical. This study aims to evaluate the accuracy, comprehensibility, and actionability of ChatGPT’s responses to common questions related to sarcopenia according to predefined criteria. Specifically, the performance of ChatGPT in areas such as sarcopenia-specific nutritional recommendations, the effectiveness of protein sources, and the role of micronutrients has been objectively analyzed. Examining ChatGPT’s capacity to provide information for specific medical conditions like sarcopenia is important for understanding the potential applications of this technology. Sarcopenia is a complex condition characterized by the loss of muscle mass and function with age, requiring an effective management strategy that combines diet, exercise, and medical approaches. In this context, AI-supported tools like ChatGPT can offer individuals quick and accessible information. However, ChatGPT’s recommendations have limitations such as lack of personalization, insufficient sensitivity to contextual details, and the risk of misinterpreting some critical clinical information. This study will also provide an opportunity to identify existing shortcomings and areas for improvement by evaluating ChatGPT’s ability to provide fundamental information about sarcopenia and respond to individual needs.

ChatGPT is an artificial intelligence tool that facilitates access to healthcare services and offers an innovative approach, particularly in areas such as nutritional advice. Users can access information at any time from different devices, providing a significant advantage for individuals living in regions with limited access to healthcare services. The platform’s 24/7 availability allows individuals to receive support quickly when needed, while reducing waiting times associated with traditional healthcare services, thereby offering a practical solution [29]. Additionally, the fact that ChatGPT is a free tool can eliminate financial barriers to accessing healthcare services. However, variations in the quality of ChatGPT’s responses have been observed; some responses may be incomplete or potentially misleading. Therefore, caution should be exercised when making decisions based solely on ChatGPT. Retraining these models with more data, especially concerning specific medical conditions like sarcopenia, can enhance personalization features and deepen contextual sensitivity. In future versions, control mechanisms developed in collaboration with clinicians or expert dietitians could provide the opportunity to detect errors in responses at an early stage. This would allow for more reliable modeling of personal risk factors, comorbid conditions, or medication interactions required by a patient-centered approach. These improvements would facilitate ChatGPT-like artificial intelligence tools in offering a holistic support mechanism not only in the field of sarcopenia but also for different clinical profiles and patient groups.

One of the strengths of this study is the use of a clearly defined scale adapted from Magruder et al. [11] for evaluating questions in the areas of sarcopenia and nutrition. This approach ensured that ChatGPT’s responses were assessed based on clearly defined criteria and scales rather than subjective judgments. Additionally, the responses were evaluated in terms of relevance, accuracy, completeness, and clarity by physicians specialized in sarcopenia and nutrition with experience in different healthcare systems. This helped reduce potential biases regarding the quality of the responses.

However, the study has some limitations. The evaluators knew that the responses to the questions were provided by ChatGPT, which may have introduced unintentional bias into the evaluation process. This situation could complicate achieving complete objectivity in assessing the responses.

## 6. Conclusions

The results of this study indicate that ChatGPT has the potential to serve as a virtual assistant that can assist patients with questions related to sarcopenia and nutrition. Additionally, it confirms ChatGPT’s potential role as a complementary resource for patient education. Future research should focus on various areas to enhance and validate ChatGPT’s benefits in sarcopenia and related nutritional guidance. Firstly, integrating individual patient data, such as body composition analysis or comorbidities, into ChatGPT’s responses could enable the provision of more personalized and effective recommendations. Furthermore, studies comparing ChatGPT’s performance with other artificial intelligence-based solutions could contribute to identifying best practices and improving the algorithm.

## Figures and Tables

**Table 1:** The 20 most frequently asked questions about sarcopenia and nutrition.

		Average Score for All Criteria	Median (Min–Max) for All Criteria	Rate of Respondents Giving a Rating of ≥4 for All Criteria, %
**Q1**	What is sarcopenia and how is it related to nutrition?	4.60	5.0 (2.0–5.0)	97.06
**Q2**	Which nutrients should I prioritize to increase or maintain my muscle mass (proteins, vitamins, minerals)?	4.54	5.0 (2.0–5.0)	97.06
**Q3**	How much daily protein do I need, and from which foods should I obtain these proteins?	4.51	5.0 (2.0–5.0)	89.71
**Q4**	Are plant-based protein sources sufficient to reduce the risk of sarcopenia, or should I necessarily consume animal proteins?	4.65	5.0 (3.0–5.0)	94.85
**Q5**	How frequently should I consume protein sources such as eggs, fish, chicken, or red meat?	4.64	5.0 (3.0–5.0)	95.59
**Q6**	Are protein powders or amino acid supplements recommended for sarcopenia? When and how should I use them?	4.48	5.0 (2.0–5.0)	90.44
**Q7**	Do vitamin D, calcium, or omega-3 fatty acids help maintain muscle mass? From which foods can I obtain these nutrients?	4.57	5.0 (3.0–5.0)	99.26
**Q8**	How should I plan the number of meals and the intervals between meals to prevent or slow down sarcopenia?	4.60	5.0 (3.0–5.0)	94.85
**Q9**	In cases of overweight or obesity, how can I lose weight healthily while preserving muscle mass?	4.58	5.0 (2.0–5.0)	95.59
**Q10**	What are the nutritional differences in age groups at high risk of sarcopenia (e.g., over 65 years old)?	4.55	5.0 (3.0–5.0)	94.85
**Q11**	What type of diet should I adopt to preserve my muscles? For example, does a Mediterranean diet benefit sarcopenia?	4.67	5.0 (3.0–5.0)	97.79
**Q12**	How do lifestyle factors (exercise, sleep, stress management) affect sarcopenia, and what is their interaction with nutrition?	4.64	5.0 (3.0–5.0)	98.53
**Q13**	When grocery shopping, which foods should I prioritize to support my muscle mass, and which should I avoid?	4.65	5.0 (2.0–5.0)	96.32
**Q14**	Can dietary supplements (e.g., creatine, BCAAs, collagen) contribute to the management of sarcopenia, or should I be cautious about them?	4.58	5.0 (2.0–5.0)	91.91
**Q15**	As a person with sarcopenia, should I seek help from a dietitian or specialist when planning my nutrition, or are general recommendations sufficient?	4.72	5.0 (2.0–5.0)	96.32
**Q16**	Are dietary approaches like intermittent fasting effective in preventing or treating sarcopenia?	4.65	5.0 (2.0–5.0)	94.85
**Q17**	Which vitamins and minerals support muscle health and are important in preventing sarcopenia (e.g., magnesium, zinc, vitamin D)?	4.65	5.0 (3.0–5.0)	94.85
**Q18**	How does appetite loss in elderly individuals affect sarcopenia, and what nutritional strategies can be applied to increase appetite?	4.68	5.0 (3.0–5.0)	99.26
**Q19**	Does limiting carbohydrate intake increase the risk of sarcopenia, or is it more important to focus on protein?	4.58	5.0 (2.0–5.0)	90.44
**Q20**	What types of foods should I consume before or after exercise, and how does this make a difference in the treatment of sarcopenia?	4.64	5.0 (2.0–5.0)	94.12

**Table 2:** Descriptive statistics of scores.

	Relevance *	Accuracy *	Clarity *	Completeness *	p ** (Cohen’s d)
All Questions					
Mean ± SD	4.72 ± 0.06	4.60 ± 0.09	4.55 ± 0.08	4.56 ± 0.15	Relevance vs. Accuracy < 0.001 (1.56) Relevance vs. Clarity< 0.001 (2.40) Relevance vs. Completeness = 0.001 (1.40) Accuracy vs. Clarity = 0.054 (0.58) Accuracy vs. Completeness = 0.642 (0.32) Clarity vs. Completeness = 0.586 (0.08)
Median (min–max)	4.71 (4.65–4.82)	4.58 (4.41–4.76)	4.55 (4.35–4.68)	4.58 (4.24–4.79)	

SD: standard deviation. * The scores given by 34 evaluators were summed, and an average score for a single question was calculated. ** Wilcoxon signed rank test.

**Table 3:** Overall agreement among evaluators.

	ICC Value	95%CI	*p*
Relevance	−0.104	−0.391; −0.287	0.684
Accuracy	0.127	−0.195; 0.493	0.208
Clarity	0.022	−0.350; 0.437	0.417
Completeness	0.569	0.324; 0.780	<0.001
Total	0.416	0.261; 0.562	<0.001

ICC: intraclass correlation coefficient. CI: confidence interval.

## Data Availability

The datasets generated and analyzed during the current study are available from the corresponding author upon reasonable request.

## References

[1] Rosenberg I.H. (1997). Sarcopenia: Origins and clinical relevance. J. Nutr..

[2] Cruz-Jentoft A.J., Bahat G., Bauer J., Boirie Y., Bruyère O., Cederholm T., Cooper C., Landi F., Rolland Y., Sayer A.A. (2019). Sarcopenia: Revised European consensus on definition and diagnosis. Age Ageing.

[3] Fielding R.A., Vellas B., Evans W.J., Bhasin S., Morley J.E., Newman A.B., van Kan G.A., Andrieu S., Bauer J., Breuille D. (2011). Sarcopenia: An undiagnosed condition in older adults. Current consensus definition: Prevalence, etiology, and consequences. J. Am. Med. Dir. Assoc..

[4] Bauer J.M., Verlaan S., Bautmans I., Brandt K., Donini L.M., Maggio M., McMurdo M.E., Mets T., Seal C., Wijers S.L. (2015). Effects of a vitamin D and leucine-enriched whey protein nutritional supplement on measures of sarcopenia in older adults, the PROVIDE study: A randomized, double-blind, placebo-controlled trial. J. Am. Med. Dir. Assoc..

[5] He J., Baxter S.L., Xu J., Xu J., Zhou X., Zhang K. (2019). The practical implementation of artificial intelligence technologies in medicine. Nat. Med..

[6] Shatte A.B.R., Hutchinson D.M., Teague S.J. (2019). Machine learning in mental health: A scoping review of methods and applications. Psychol. Med..

[7] Kung T.H., Cheatham M., Medenilla A., Sillos C., Leon L., Elepaño C., Madriaga M., Aggabao R., Diaz-Candido G., Maningo J. (2023). Performance of ChatGPT on USMLE: Potential for AI-Assisted Medical Education Using Large Language Models. PLoS Digit. Health.

[8] Miner A.S., Milstein A., Schueller S., Hegde R., Mangurian C., Linos E. (2016). Smartphone-based conversational agents and responses to questions about mental health, interpersonal violence, and physical health. JAMA Intern. Med..

[9] Laranjo L., Dunn A.G., Tong H.L., Kocaballi A.B., Chen J., Bashir R., Surian D., Gallego B., Magrabi F., Lau A.Y.S. (2018). Conversational agents in healthcare: A systematic review. J. Am. Med. Inform. Assoc..

[10] Gilson A., Safranek C.W., Huang T., Socrates V., Chi L., Taylor R.A., Chartash D. (2023). How Well Does ChatGPT Do When Taking the Medical Licensing Exams? The Implications of Large Language Models for Medical Education and Knowledge Assessment. JMIR Med. Educ..

[11] Magruder M.L., Rodriguez A.N., Wong J.C.J., Erez O., Piuzzi N.S., Scuderi G.R., Slover J.D., Oh J.H., Schwarzkopf R., Chen A.F. (2024). Assessing ability for ChatGPT to answer total knee arthroplasty-related questions. J. Arthroplast..

[12] Bauer J., Biolo G., Cederholm T., Cesari M., Cruz-Jentoft A.J., Morley J.E., Phillips S., Sieber C., Stehle P., Teta D. (2013). Evidence-based recommendations for optimal dietary protein intake in older people: A position paper from the PROT-AGE Study Group. J. Am. Med. Dir. Assoc..

[13] Koo T.K., Li M.Y. (2016). A Guideline of Selecting and Reporting Intraclass Correlation Coefficients for Reliability Research. J. Chiropr. Med..

[14] Shrout P.E., Fleiss J.L. (1979). Intraclass correlations: Uses in assessing rater reliability. Psychol. Bull..

[15] Wei Q., Yao Z., Cui Y., Wei B., Jin Z., Xu X. (2024). Evaluation of ChatGPT-generated medical responses: A systematic review and meta-analysis. J. Biomed. Inform..

[16] Kirk D., Van Eijnatten E., Camps G. (2023). Comparison of answers between ChatGPT and human dieticians to common nutrition questions. J. Nutr. Metab..

[17] Chatelan A., Clerc A., Fonta P.A. (2023). ChatGPT and future artificial intelligence chatbots: What may be the influence on credentialed nutrition and dietetics practitioners?. J. Acad. Nutr. Diet..

[18] Papastratis I., Stergioulas A., Konstantinidis D., Daras P., Dimitropoulos K. (2023). Can ChatGPT provide appropriate meal plans for NCD patients?. Nutrition.

[19] Sallam M. (2023). ChatGPT utility in healthcare education, research, and practice: Systematic review on the promising perspectives and valid concerns. Healthcare.

[20] Jiang F., Jiang Y., Zhi H., Dong Y., Li H., Ma S., Wang Y., Dong Q., Shen H., Wang Y. (2017). Artificial intelligence in healthcare: Past, present and future. Stroke Vasc. Neurol..

[21] Topol E.J. (2019). High-performance medicine: The convergence of human and artificial intelligence. Nat. Med..

[22] Yapar D., Demir Avcı Y., Tokur Sonuvar E., Eğerci Ö.F., Yapar A. (2024). ChatGPT’s potential to support home care for patients in the early period after orthopedic interventions and enhance public health. Jt. Dis. Relat. Surg..

[23] Esteva A., Robicquet A., Ramsundar B., Kuleshov V., DePristo M., Chou K., Cui C., Corrado G., Thrun S., Dean J. (2019). A guide to deep learning in healthcare. Nat. Med..

[24] Kalla D., Smith N. (2023). Study and analysis of chat GPT and its impact on different fields of study. Int. J. Innov. Sci. Res. Technol..

[25] Atwal K. (2024). Artificial intelligence in clinical nutrition and dietetics: A brief overview of current evidence. Nutr. Clin. Pract..

[26] Luo J., Li T., Wu D., Jenkin M., Liu S., Dudek G. (2024). Hallucination Detection and Hallucination Mitigation: An Investigation. arXiv.

[27] Ji Z., Lee N., Frieske R., Yu T., Su D., Xu Y., Ishii E., Bang Y.J., Madotto A., Fung P. (2023). Survey of hallucination in natural language generation. ACM Comput. Surv..

[28] You M., Chen X., Liu D., Lin Y., Chen G., Li J. (2024). ChatGPT-4 and wearable device assisted Intelligent Exercise Therapy for co-existing Sarcopenia and Osteoarthritis (GAISO): A feasibility study and design for a randomized controlled PROBE non-inferiority trial. J. Orthop. Surg. Res..

[29] Sharma S., Pajai S., Prasad R., Wanjari M.B., Munjewar K.P., Sharma R., Pathade A. (2023). A critical review of ChatGPT as a potential substitute for diabetes educators. Cureus.

